# Efficacy of exercise interventions for executive function in children with ADHD: What is the current verdict?

**DOI:** 10.1038/s41390-025-04646-1

**Published:** 2025-11-25

**Authors:** Nicole E. Logan, Michelle Lim

**Affiliations:** 1 The University of Rhode Island, Department of Kinesiology, Kingston, RI US; 2 The University of Rhode Island, Interdisciplinary Neuroscience Program, Kingston, RI US; 3 The University of Rhode Island, George and Anne Ryan Institute for Neuroscience, Kingston, RI US

The systematic review and Bayesian meta-analysis by Pan, Zheng & He (2025)^1^ assesses the impact of exercise on executive function in children with ADHD. Executive function was categorized into three domains: working memory, inhibitory control, and cognitive flexibility. Using a Bayesian dose-response framework, the meta-analysis incorporated an estimate of exercise dose to model the non-linear relationship between exercise and executive function. To achieve this, Pan et al. quantified metabolic equivalents of task (METs) from 33 studies, spanning acute and chronic exercise interventions in children with ADHD, to use as an estimate for exercise dose. Their study highlighted that exercise-induced effects on executive function in children with ADHD were dose-dependent.

The key finding of the study was the identification of domain-specific dose-response patterns for executive function. To do this, Pan et al. novelly quantified the exercise dose from 33 studies using age-specific METs from the 2017 Youth Compendium of Physical Activity.^2^ Specifically, for acute sessions, exercise dose was calculated by multiplying the MET value of a specified youth activity by the duration of the exercise, expressed as MET-min/session. For chronic interventions, a weekly exercise dose was calculated as the product of session duration and frequency, which produced a metric expressed in MET-min/week. It is important to note that METs from the Youth Compendium of Physical Activity (MET_y_) range from low to high depending on the intensity of activities. For example, quietly lying down (1.1 MET_y_) produces a smaller MET value than standing (1.9 MET_y_), which requires slightly more metabolic effort. MET_y_ values typically range from 1.1-10, but more intense activities, such as swimming (10.6 MET_y_) and running (13.2 MET_y_) can exceed that general range. This is an important consideration when interpreting estimated exercise doses. Pan et al. presented either MET-min/session, or MET-min/week, which are much larger values than typical MET_y_ values. The approach to quantify METs over a single session or a week provided a standardized metric for comparing intervention doses across studies. Their results suggested that acute exercise estimated at 130 MET-min/session contributed toward greater inhibitory control, but the optimal dose for working memory was higher at 170 MET-min/session, and cognitive flexibility required a dose of 270 MET-min/session. Long-term interventions required substantially higher weekly doses to achieve optimal effects, such as around 2500 MET-min/week for inhibitory control, 1300 MET-min/week for working memory, and 1100 MET-min/week for cognitive flexibility. A major strength of the current study is the standardized metric across interventions, allowing for the inclusion of more studies in the meta-analysis. However, as MET_y_ values represent the metabolic efforts of the pediatric body during different activities (typically ranges from 1.1 to 13.2), the novel MET-estimates value might be limited in its real-world implications, as no single activity represents the range of MET-estimates provided in Pan et al. (e.g., 130 MET-min/session or 2500 MET-min/week). Another consideration for interpreting the results of Pan et al. is that the acute and chronic MET-estimates were calculated and then grouped into discrete dose categories to facilitate the network meta-analysis. Future studies should consider the MET-estimate approach with continuous variables in order to fully understand the potential of using this approach as a conventional dose estimate.

Overall, the findings from Pan et al. challenge the assumption of uniform cognitive benefits from exercise and underscore the importance of considering both cognitive domain and exercise dose when designing interventions. Recently, other meta-analyses have identified that children with ADHD might uniquely benefit from aerobic physical activities that also require a cognitive engagement component.^3^ Exercise bouts that are combined with cognitive elements, such as multi-component ball sports (e.g., basketball, soccer) and mind-body activities (e.g., karate), are generally thought to have a greater positive effect on cognitive performance for children with ADHD.^4–6^ While the use of MET-estimates provides a dose-response curve, the approach does not include information on types of activities that might be uniquely beneficial for children with ADHD. Future research could extend this work by examining MET-estimates as continuous variables and exploring how activities of varying cognitively engaging elements interact with cognitive outcomes. This framework would lead to a more nuanced understanding of the dose-response relationships and help refine exercise intervention strategies for ADHD.

What are the implications of this review? The meta-analysis by Pan et al. proposes that their findings could inform future neuroimaging and multimodal physiological studies to investigate dose threshold and saturation effects on brain network dynamics. While this is a valuable direction for pediatric ADHD research, it is important to recognize that behavior and cognitive outcomes of school-aged children are not necessarily contingent on neuroimaging validation. ADHD is fundamentally a behavioral disorder,^7^ and executive functions in school-aged children are often best captured through behavioral assessments, not necessarily through neuroimaging techniques. Although structural and functional neuroimaging techniques can offer valuable insights into the neural correlates of cognitive function, these measures are intertwined with neurodevelopmental processes and cannot be easily isolated from behavior.^8^ During childhood and adolescence, brain maturation and behavioral adaptation are deeply interconnected, making it difficult to determine whether observed neural changes are causes, consequences, or correlates of cognitive improvement.^9^ Moreover, neuroimaging techniques, such as electroencephalography (EEG) and/or magnetic resonance imaging (MRI) data may moderate, but do not always define, cognitive outcomes. Future research may benefit from interdisciplinary approaches that blend behavioral theories of ADHD and neuroimaging techniques.

What should future research consider? Among the wider literature, there is a tendency for researchers to either use a clinical diagnosis of ADHD to create comparative groups or to approach analyses using continuous ADHD symptoms. Both approaches offer valuable insight; however, ADHD itself is not a unified condition, thus a single diagnosis or a report of total symptoms does not fully represent the condition. Children with ADHD can be diagnosed with either the inattentive subtype, the hyperactivity-impulsivity subtype, or the combined subtype. Likewise, measures that rely on symptom ratings can also reflect these three subtypes, yet most research considers ADHD as a single condition, presumably for reasons of maintaining statistical power and/or to identify broader patterns. However, this approach may overlook meaningful individual differences between the ADHD subtypes. This is an important consideration for clinical implications; however, it is also meaningful to consider how ADHD relates to executive function. Emerging evidence suggests that executive function deficits are not evenly distributed across the ADHD subtypes. Children with the inattentive subtype might experience greater deficits in executive functions such as cognitive processing, inhibitory control, and working memory; meanwhile, children with the hyperactivity-impulsivity subtype might experience greater difficulty with emotion regulation, reward processing, delay gratification, and making flexible decisions under emotional influence.^10^ These distinctions raise the question of whether exercise interventions, which are increasingly used to support executive function, should be expected to yield uniform benefits across all ADHD subtypes. Consequently, this consideration also extends to a wider mechanistic question within our field. Specifically, exercise neuroscience researchers are greatly invested in the domain-specific or global features of the exercise intervention itself (e.g., frequency, intensity, time, type, total duration), the executive functions affected by exercise (e.g., attention, inhibition, working memory, cognitive flexibility), and the neural correlates of these relationships (e.g., brain structure, function, electrophysiology). If different exercise modalities recruit different executive function domains, then it follows that children with different ADHD presentations may differentially respond to exercise. Yet, most meta-analyses do not stratify by subtype, leaving a gap in our understanding of how to tailor interventions more precisely. It would be valuable to understand whether children with different ADHD profiles respond differently to specific exercise modalities, and whether such differences moderate the cognitive effects of exercise in ways that could inform more targeted, developmentally appropriate strategies to improve executive function.

In summary, the systematic review and Bayesian meta-analysis by Pan et al. provides a novel dose-response perspective using MET-estimates on the efficacy of using exercise interventions to improve executive functions in children with ADHD. While this and several other recent meta-analyses have significantly contributed to the field, several methodological, theoretical, and practical questions remain. Future work should seek to complement the current dose-response patterns by (i) describing meaningful physical activity doses, (ii) performing large-scale randomized controlled trials that include cognitively engaging forms of exercise, and (iii) isolating the effects of ADHD subtypes and symptoms on the exercise-related benefits to behavioral and neural correlates of executive function.
